# The VNTR Polymorphism of the DC-SIGNR Gene and Susceptibility to HIV-1 Infection: A Meta-Analysis

**DOI:** 10.1371/journal.pone.0042972

**Published:** 2012-09-05

**Authors:** Hui Li, Xiao-Min Yu, Jia-Xin Wang, Ze-Hui Hong, Nelson Leung-Sang Tang

**Affiliations:** 1 Department of Genetics and Developmental Biology, Southeast University School of Medicine, Nanjing, China; 2 Department of Biochemistry, Southeast University School of Medicine, Nanjing, China; 3 The Key Laboratory of Developmental Genes and Human Disease, Ministry of Education, Southeast University, Nanjing, China; 4 Laboratory for Conservation and Utilization of Bio-Resource and Key Laboratory for Microbial Resources of the Ministry of Education, Yunnan University, Kunming, China; 5 Li Ka Shing Institute of Health Sciences and Department of Chemical Pathology, The Chinese University of Hong Kong, Shatin, Hong Kong, Shatin, Hong Kong; 6 Departments of Chemical Pathology, Faculty of Medicine, The Chinese University of Hong Kong, Shatin, Hong Kong; 7 Functional Genomics and Computational Statistics, Shenzhen Research Institute, CUHK, ShenZhen, China; Baylor College of Medicine, United States of America

## Abstract

**Background:**

Dendritic cell-specific intercellular adhesion molecule-3-grabbing nonintegrin related (DC-SIGNR) can bind to the human immunodeficiency virus-1 (HIV-1) gp120 envelope glycoprotein and is thus important for the host-pathogen interaction in HIV-1 infection. Studies of the association between the variable number tandem repeat (VNTR) polymorphism of the DC-SIGNR gene and HIV-1 susceptibility have produced controversial results.

**Methods and Findings:**

We conducted a meta-analysis of the data contained in the literature to clarify these findings. In total, 10 studies consisting of 2683 HIV-1 patients and 3263 controls (2130 healthy controls and 1133 HIV-1 exposed but seronegative (HESN) controls) were included. Odds ratios (ORs) with 95% confidence intervals (95% CIs) were assessed in the main analyses. Further stratified analyses by ethnicity and sample size were performed. By dividing the controls into two groups, healthy controls and HIV-1 exposed but seronegative (HESN) controls, we explored different genetic models to detect any association between the VNTR polymorphism and predisposition to HIV-1 infection. The results showed that the 5-repeat allele carriers (OR = 0.84, 95% CI = 0.73–0.96) and the 5/5 homozygous (OR = 0.68, 95% CI = 0.50–0.93) had significantly reduced risk when using the HIV-1 exposed but seronegative (HESN) as controls. The stratified analyses by ethnicity and sample size confirmed these findings. However, a low to moderate degree of heterogeneity was also found across studies.

**Conclusions:**

Our findings demonstrate that the VNTR polymorphism of the DC-SIGNR gene is associated with a moderate effect on host susceptibility to HIV-1 infection. Similar to the 32-bp deletion in the chemokine receptor-5 gene (CCR5Δ32), the DC-SIGNR VNTR 5-repeat allele might have a role in resistance to HIV infection, particularly in Asian populations.

## Introduction

The incidence of acquired immunodeficiency syndrome (AIDS) has increased over the past few decades. Up to now, nearly 34 million people suffered from human immunodeficiency virus-1 (HIV-1) infection, and an estimated 2.7 million people were newly infected with the virus in 2010 (http://www.who.int/features/factfiles/hiv/facts/en/index3.html). However, the natural course of HIV-1 infection and the susceptibility to infection after exposure are highly heterogeneous among individuals [1], [2]. Despite of high-risk behavior and/or multiple exposures to HIV-1, some individuals remained seronegative, or uninfected. These individuals may have a different course of progression to AIDS and may have different clinical outcomes. It is a common observation that some infected individuals became symptomatic within 2–3 years while others remained asymptomatic for more than 10–15 years [3]. Last year, NIH published a conference report about definition of HIV-exposed seronegative (HESN) individuals. It is now a consensus to define HESN from several group of individuals who are at high risk of exposure which include : (1) the commercial sex workers, (2) people with hemophilia, (3) discordant couples, (4) intravenous drug users, and (5) mother-to-child transmission [4].

In the past few years, many studies revealed that host immunogenetics, including genetic polymorphisms, play important roles in host resistance to HIV-1 infection and predict different progressions to AIDS after infection [5]–[7]. Polymorphisms of certain chemokines and chemokine receptors have been reported to play important roles in individual variability in response to HIV-1/AIDS. The most investigated variation is the 32-bp deletion in the chemokine receptor-5 (CCR5 Δ32) gene, which was shown to confer resistance to HIV-1 infection in homozygous carriers, and its role has been investigated in a clinical context [8], [9]. Similar to CCR5, DC-SIGNR is potentially an important gene affecting host susceptibility to HIV-1 infection and disease progression. DC-SIGN (dendritic cell-specific intercellular adhesion molecule-3-grabbing nonintegrin) is able to bind the HIV-1 gp120 surface glycoprotein with high affinity and is able to enhance trans-infection of T cells by HIV-1 [10]. DC-SIGNR is a homolog of DC-SIGN with 77% amino acid identity and preferential expression in liver and lymph node epithelial cells. DC-SIGNR functions similarly to DC-SIGN in capturing HIV-1 and enhancing HIV-1 infection of T cells [11].

Both DC-SIGN and DC-SIGNR are organized into 3 domains: (1) an N-terminal cytoplasmic region followed by a transmembrane domain, (2) a neck-region containing a variable number tandem repeat (VNTR) of a conserved 23 amino acid sequence, and (3) a C-terminal extracellular domain with a C-type carbohydrate recognition domain (CRD) involved in pathogen binding [12]. While the CRD forms complicated carbohydrates with high mannose-containing ligands, the neck-region is essential for lectin tetramerization and influences the capability of CRD to interact with pathogens such as HIV-1. Because the VNTR of the neck-region in DC-SIGNR is highly polymorphic, ranging from 3 to 9 repeats in worldwide populations, the polymorphism has been widely studied with regard to host genetic predisposition to HIV-1 infection [13]–[23]. However, the results are controversial and inconclusive, most likely because of the different ethnic populations used in the different studies or lacking the statistical power in any individual study to produce a reliable conclusion. Thus, a comprehensive analysis is critical.

In this study, a meta-analysis was performed to investigate the association between the VNTR polymorphism of the DC-SIGNR gene and host susceptibility to HIV-1 infection. We also performed a stratified analysis by ethnicity and sample size to explore the variation in the relationship between VNTR polymorphism and HIV-1 infection risk among different ethnic populations.

## Materials and Methods

### Literature Search

We performed a literature search in PubMed, MEDLINE, Embase, Wanfang Database and Weipu Database for studies published through April 2011 to use in this meta-analysis. The key terms were “DC-SIGNR or CLEC4M or CD209L or L-SIGN or CD299 or DC-SIGN2”, “HIV-1”, and “polymorphism” in various combinations. The search was limited to articles published in English and Chinese. We also manually searched the references matching the above criteria to identify additional studies.

### Inclusion and Exclusion Criteria

The eligible investigations met the following criteria: (1) the studies were case-control designed to explore the association between VNTR polymorphism in the DC-SIGNR gene and HIV-1 susceptibility; (2) the studies provided data on the distribution of VNTR polymorphism in the case-control population; (3) the studies were published in the English or Chinese languages. Studies were excluded if they did not contain enough data for meta-analysis, if they were abstracts or reviews, or if they were duplicated within other included studies.

### Data Extraction

Two authors perused the papers and extracted the data according to the above inclusion and exclusion criteria. The following characteristics were collected from the eligible studies: first author, year of publication, country of studied population, ethnicity, sample size of the cohorts, number of HIV-1 patients, number of normal healthy controls, number of HIV-1 exposed but seronegative (HESN) controls, and the distribution of the DC-SIGNR VNTR genotype among case and control groups.

### Statistical Analyses

ORs with 95% CI were calculated to assess the association between VNTR polymorphism in the DC-SIGNR gene and HIV-1 susceptibility. In the primary analysis, an allelic association test was performed for the prevalent alleles, which were (1) the 7-repeat allele, (2) the 5-repeat allele, (3) the 6-repeat allele and (4) the 9-repeat allele. These alleles represented the four most prevalent alleles in human population. To correct for multiple testing for these 4 alleles, a Bonferroni correction was also applied to determine the corrected p-value (p-value corrected). After that it was found that 5-repeat allele had a protective effect. An exhaustive round of post-hoc analysis was carried out to identify if any particular genetic model was in operation. In this post-hoc analysis, the pooled ORs were performed between HIV-1 patients and control groups in the following genetic models: (1) the homozygote proportion [24], [25], (2) the 7/7 genotype vs. the other genotypes, (3) the 5/5 genotype vs. the other genotypes, (4) the 5/7 genotype vs. the other genotypes, (5) the 6/7 genotype vs. the other genotypes, (6) the 7/9 genotype vs. the other genotypes. As it is an explorative attempt to delineate the mode of operation account for the protective effect in the genetic test, no Bonferroni correction was carried out in this stage. In all the genetic models, the types of controls were used: (1) healthy normal or population controls and (2) HIV-1 exposed but seronegative (HESN) controls (see the Table S1 for detailed breakdown). Because the remaining rare genotypes and alleles of the VNTR locus have negligible frequencies in the cohorts, their comparisons were not conducted in this analysis. Stratified analyses were performed by ethnicity and sample size.

Heterogeneity across the publications was assessed with the Cochran's <chi>^2^ test (Q-test) [26], and *p*<0.10 was considered statistically significant. The I^2^ test was also conducted to evaluate heterogeneity between studies. A high heterogeneity is considered present when I^2^>50% and much higher when I^2^>75% [27]. The pooled OR was calculated by a fixed effect model (using the Mantel-Haenszel method) or a random effect model (using the DerSimonian-Laird method) according to the heterogeneity among studies [28]. The statistical significance of OR was analyzed by the Z test, and *p*<0.05 was considered statistically significant. Publication bias was evaluated by Egger's and Begg's tests and was considered significant if *p*<0.05 [29], [30]. Sensitivity analysis was performed by sequentially excluding individual studies to assess the stability of the results.

Statistical analyses were performed using the Revman 5.1 software and STATA 10.0 software. The *p* value was two-tailed and was considered to be statistically significant if its value was <0.05.

## Results

### Study Characteristics

**Figure 1:**
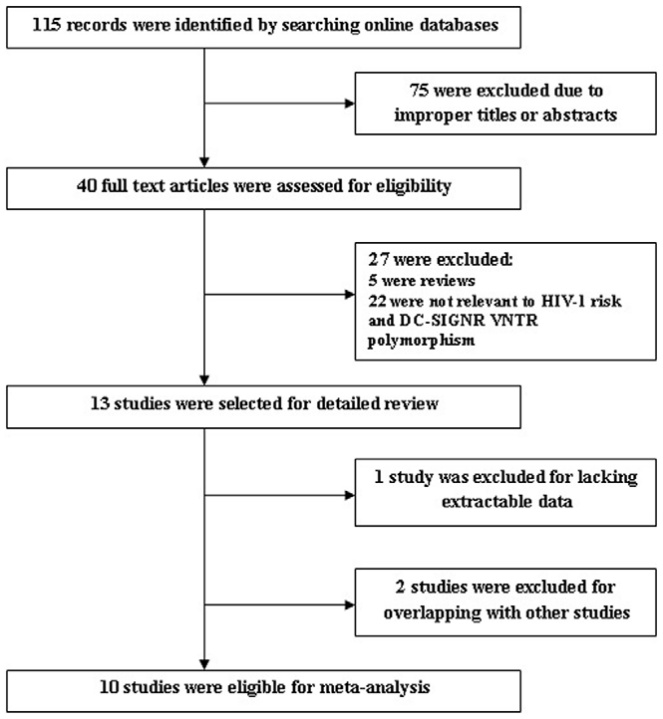
Flow diagram of the study selection for the meta-analysis.

As shown in Figure 1, a total of 115 results were identified after an initial search from the selected electronic databases. After screening the titles and abstracts, 40 articles were selected for further review. Among them, five reviews were excluded; 22 studies were excluded for not referring to the association between the DC-SIGNR VNTR polymorphism and HIV-1 susceptibility. Finally, one study was excluded because it lacked extractable data; and two studies were excluded because the data or sample sets overlapped with another one. Thus, a total of 10 studies were suitable for meta-analysis [13]–[18], [20]–[23]. Among them, seven studies were conducted with Asians [13], [16]–[18], [20], [21], [23], two were conducted with European-Americans [14], [15] and one was conducted with Africans [22]. Two studies only included subjects of HIV-1 patients and HIV-1 exposed but seronegative (HESN) controls [21], [22]. Three studies included subjects of HIV-1 patients and healthy controls [15], [18], [20], while the remaining five studies included subjects of HIV-1 patients, HIV-1 exposed but seronegative (HESN) controls, and healthy controls [13], [14], [16], [17], [23]. The total number of samples involved in the 10 eligible studies was 5946, which included 2683 HIV-1 patients and 3263 controls (2130 healthy controls and 1133 HIV-1 exposed but seronegative (HESN) controls). The characteristics of each study are listed in Table 1.

**Table 1:** Characteristics of the studies included in the meta-analysis.

First author	Year	Country	Ethnicity	Cases (HIV-1 positive)	Healthy Control (HIV-1 negative)	HESN Control (HIV-1 negative)
Liu H	2006	USA	European-American	801	698	217
Lichterdeld M	2003	Germany	European-American	540	134	
Rathore A	2008	India	Asian	168	262	47
Chaudhary O	2008	India	Asian	100	100	150
Wichukchinda N	2007	Thailand	Asian	305	290	102
Xu LJ	2010	China	Asian	144	173	
Zhao J	2008	China	Asian	88	384	
Wang H	2008	China	Asian	345		468
Wang XH	2010	China	Asian	101	89	52
Boily-Larouche G	2009	Zimbabwe	African	91		99

Note: HESN, HIV-exposed seronegative individuals.

### Meta-analysis

After pooling the data from the 10 studies for meta-analysis, the results were calculated according to different genetic models. For each genetic model, two comparisons were carried out separately using either the healthy samples or the HIV-1 exposed but seronegative samples (HESN) as controls.

**Figure 2:**
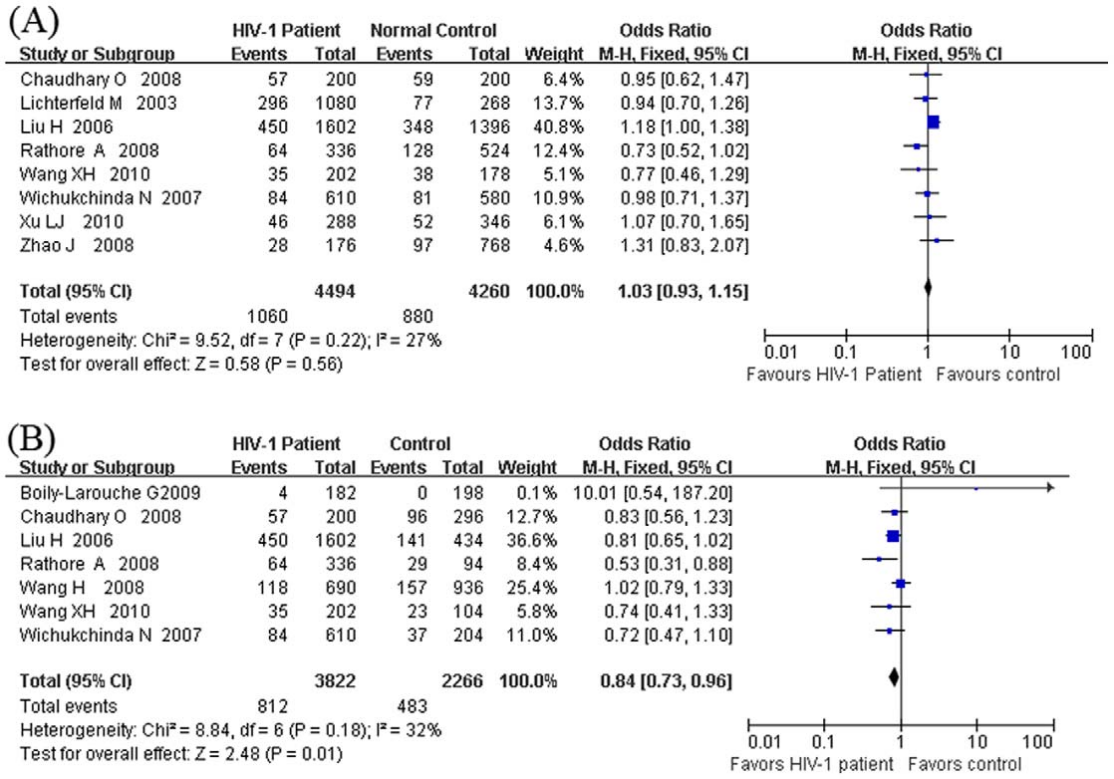
Forest plot of the association between HIV-1 infection and the DC-SIGNR VNTR polymorphism under the allelic risk model of 5-repeat allele vs. other alleles. (A)By using the healthy samples as controls, the comparison was carried out between the HIV-1 patients and healthy samples; (B) By using the HIV-1 exposed but seronegative (HESN) samples as controls, the comparison was carried out between the HIV-1 patients and the HIV-1 exposed but seronegative (HESN) samples. Note: A Bonferroni correction for multiple testing of 4 was applied to get a corrected P value in this primary analysis. The association between 5-repeat allele had a corrected P value of 0.05.

In the primary analysis for allelic risk model of the 5-repeat allele vs. the other alleles, using the healthy individuals as controls, the value of X^2^ was 9.52 with 7 degrees of freedom, and *p* was 0.22 in a fixed effect model (Figure 2A). The I^2^ was 27%, suggesting a low to moderate heterogeneity. The fixed effect model was applied to synthesize the data. Overall, the OR was 1.03 (95% CI = 0.93–1.15) and was not significant (Figure 2A). On the other hand, using the HIV-1 exposed but seronegative (HESN) samples as controls, the value of X^2^ was 8.84 with 6 degrees of freedom, and *p* was 0.18 (Figure 2B). The I^2^ was 32%, suggesting a moderate heterogeneity. Then the fixed effect model was applied to synthesize the data and the pooled OR was calculated. The pooled OR value was significant at 0.84 (95% CI = 0.73–0.96, *p* = 0.01, Figure 2B). The Bonferroni corrected P-value was 0.05. These results suggested that the 5-repeat allele carriers tended to be associated with resistance to HIV-1 infection in HIV-1 exposed seronegative (HESN) individuals.

**Figure 3:**
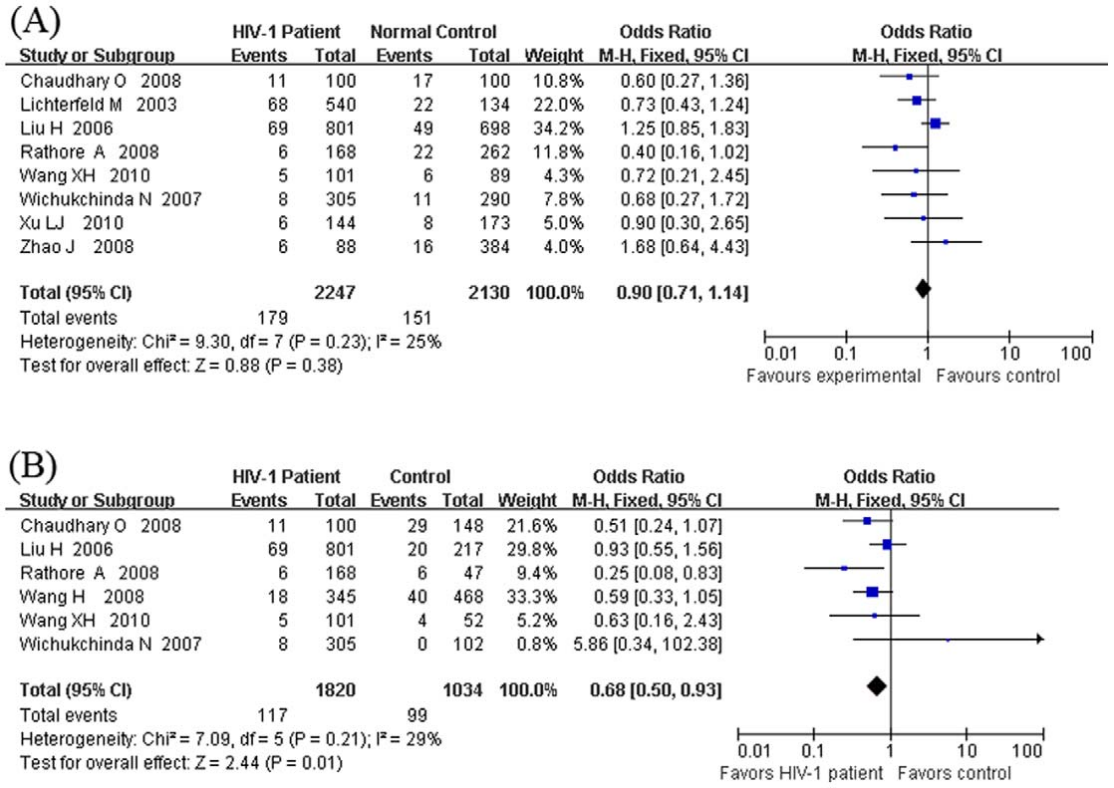
Forest plot of the association between HIV-1 infection and the DC-SIGNR VNTR polymorphism under the genotypic risk model of 5/5 vs. other genotypes. (A)By using the healthy samples as controls, the comparison was carried out between the HIV-1 patients and healthy samples; (B) By using the HIV-1 exposed but seronegative (HESN) samples as controls, the comparison was carried out between the HIV-1 patients and the HIV-1 exposed but seronegative (HESN) samples.

In the genetic models, we analyzed the heterogeneity of 5/5 vs. the other genotypes by using the healthy subjects as controls. The results indicated a low heterogeneity (X^2^ = 9.30, I^2^ = 25%, *p* = 0.23) (Figure 3A). Next, we pooled the 8 studies under the fixed effect model. The pooled OR was 0.90 (95% CI = 0.71–1.44, *p* = 0.38) (Figure 3A). Then, we analyzed the heterogeneity of 5/5 vs. the other genotypes by using the HIV-1 exposed but seronegative samples subjects (HESN) as controls (Figure 3B). The results indicated a low to moderate heterogeneity (X^2^ = 7.09, I^2^ = 29%, *p* = 0.21). The fixed effect model was then used to synthesize the data. Overall, the pooled OR was significant at 0.68 (95% CI = 0.50–0.93, *p* = 0.01) (Figure 3B). These results suggested that the 5/5 homozygous genotype showed a significantly reduced risk of HIV-1 infection in HIV-1 exposed seronegative individuals (HESN) but not in healthy individuals. A summary of the results of all the comparisons with different genetic models was listed in Table S1.

**Figure 4:**
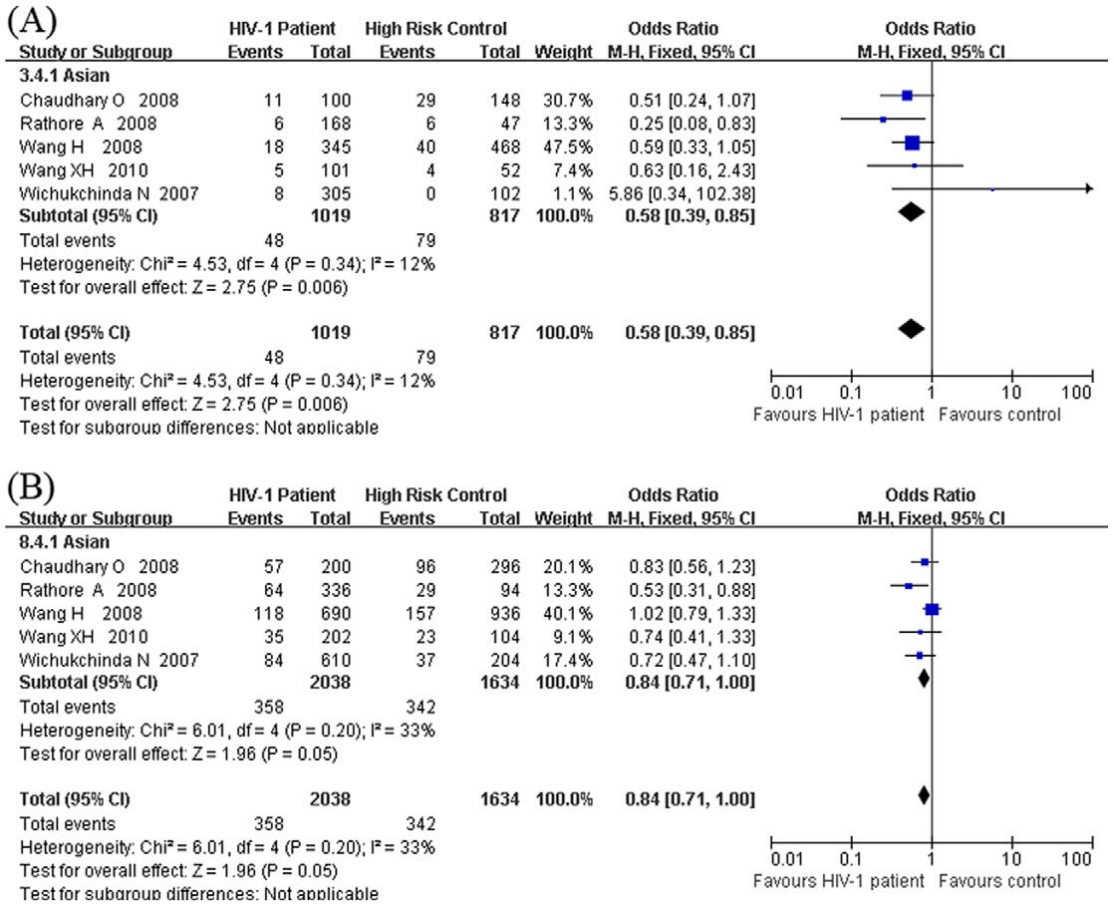
Forest plot of the association between HIV-1 infection and the DC-SIGNR VNTR polymorphism carried out in Asian populations. (A) By using the HIV-1 exposed but seronegative (HESN) samples as controls, the comparison was carried out between the HIV-1 patients and the HIV-1 exposed but seronegative (HESN) samples under the genotypic risk model of 5/5 vs. other genotypes. (B) By using the HIV-1 exposed but seronegative (HESN) samples as controls, the comparison was carried out between the HIV-1 patients and the HIV-1 exposed but seronegative (HESN) samples under the allelic risk model of 5-repeat allele vs. other alleles.

We also performed stratified analyses by ethnicity and sample size to explore potential sources of heterogeneity and examine the relationship between the DC-SIGNR VNTR polymorphism and susceptibility to HIV-1 infection. The results are also summarized in Table S2. Most of the results of the stratified analyses were consistent with the main analysis. For ethnicity, the studies were divided into two subgroups, one subgroup of Asian descendants (7 studies) and the other subgroup of European-American descendants (2 studies). The resistance to HIV-1 infection found among 5/5 homozygous subjects was predominantly due to the HIV-1 exposed seronegative (HESN) subjects of the Asian population (OR = 0.58, 95% CI = 0.39–0.85, *p* = 0.006, *P*heterogeneity = 0.34 and I^2^ = 12%) (Figure 4A). The 5-repeat allele presented the trend of having the protective effect among Asian (OR = 0.84, 95% CI = 0.71–1.00, *p* = 0.05, *P*heterogeneity = 0.20 and I^2^ = 33%), though the *p* value was marginal (Figure 4B). This result also showed that the sample size of the Asian population in present study was not large enough to achieve enough statistical power to obtain significant observations. As there was only one study performed with African and European-Americans involving HIV-1 patients and HIV-1 exposed seronegative (HESN) controls, the stratified analyses in these populations were not performed.

For sample size, the studies were stratified into two subgroups, one comprising studies with more than 200 subjects and one with fewer than 200 subjects. In these subgroups, significantly reduced HIV-1 infection of 5/5 homozygous (OR = 0.69, 95% CI = 0.50–0.94, and *p* = 0.02) and 5-repeat allele carriers (OR = 0.82, 95% CI = 0.68–0.98, and *p* = 0.03) was found in the subgroup with a sample size of more than 200 subjects, but not in the subgroup with fewer than 200 subjects (Table S2).

### Publication Bias

Publication bias was assessed by Begg's test and Egger's test (data not shown) in the total population and all the subgroups. For the genotypic risk model, 5/5 vs. other genotypes, in which the HIV-1 exposed but seronegative samples (HESN) subjects were used as controls, both the Begg's test (*p* = 0.462) and the Egger's test (*p* = 0.215) did not present any significantly statistical evidence of publication bias. For the allelic risk model of 5-repeat allele vs. other alleles, in which the HIV-1 exposed but seronegative samples (HESN) subjects were used as controls, the Begg's test (*p* = 0.26) and the Egger's test (*p* = 0.161) also showed no evidence of publication bias. Moreover, evaluations of publication bias for the other genetic models did not reveal significant results (data not shown).

### Sensitivity Analysis

Sensitivity analysis was performed by deleting one study at one time to assess the stability of the pooled ORs. None of the corresponding pooled ORs was statistically changed, implying the stability of the results.

## Discussion

A potential role of host genetic factors in the predisposition to HIV-1 infection has been suggested by different reports [8], [31], [32]. DC-SIGNR serves as an HIV-1 ligand to facilitate HIV-1 virion infection into adjacent CD4+T cells in trans and has been the subject of many recent studies. The VNTR polymorphism in its neck-region was found to be associated with host susceptibility to HIV-1 infection, but the conclusions were controversial.

In the present study, we performed a meta-analysis of 10 eligible studies with 2683 HIV-1 patients and 3263 controls to elucidate the relationship between the VNTR polymorphism and HIV-1 infection risk. The strength of the present analysis is based on the accumulation of published data that provides enough information to generate a more precise conclusion. By using different genetic models, we could preliminarily estimate the effect of the allele frequency, genotype frequency, and homozygous proportion. The results indicated that the 5/5 homozygous genotype was associated with resistance to HIV-1 infection. The protective effect was most predominant in HIV-1 exposed seronegative (HESN) individuals. The stratified analyses by ethnicity and sample size confirmed these findings. Thus, it is important to emphasize that in studies concerning HIV-1 susceptibility, taking samples from HIV-1 exposed seronegative (HESN) individuals as controls may be more powerful than taking random healthy individuals as controls. In future studies, collecting both HIV-1 exposed seronegative individuals (HESN) and random healthy individuals as controls would be helpful for clarifying the relationship between candidate genes and host susceptibility to HIV-1 infection. But under the definition and criteria of HIV-1 exposed seronegative individuals (HESN), it is still a heterogeneous group. A workshop sponsored by NIH was conducted in 2011 and a consistent definition and criteria were drawn in the term of HIV-exposed seronegative (HESN) individuals [4]. In the following studies, the collection of the HESN as controls should strictly obey these definitions, which would be helpful in explaining natural HIV-1 protection in individuals exposed to HIV-1 who remain seronegative or demonstrate resistance to infection.

Pooled data analysis by ethnicity was only performed in Asians since only one study involving HIV-1 patients and HIV-1 exposed seronegative controls (HESN) was separately performed in European-Americans and Africans. More studies on these populations are needed in future works, which would help us better understand the relationship between the VNTR polymorphism and HIV-1 infection risk in different ethnic populations.

Our findings are consistent with the study by Rathore et al. [16] but are different from other reports [14], [15], [17]. The potential explanation for the discrepancy may be the limited sample number included in the single study and the relative impact of this polymorphism in the different populations. As shown, the associations were only confirmed in stratified analyses by combining studies with a sample size greater than 200 subjects, which emphasized the importance of having sufficient power with a large enough sample size and the requirement of further large-scale analysis.

Although the function of the association between the DC-SIGNR VNTR polymorphism and host susceptibility to HIV-1 infection has not been fully explored, outcomes of this meta-analysis suggest that the DC-SIGNR VNTR polymorphism had an impact on host susceptibility to HIV-1 infection. A study by Xu et al. [18] showed that patients carrying the 5-repeat DC-SIGNR allele had significantly lower HIV-RNA levels compared with those observed in patients with the 7- and 9-repeat DC-SIGNR alleles, though they did not observe that the different genotypes/alleles were associated with CD4+ T cell numbers. *In vitro* studies also demonstrated that DC-SIGNR with equal to or less than 5-repeat alleles displayed unstable homozygous or heterozygous DC-SIGNR aggregates [33] accompanied by changes in affinity for HIV-1gp120 glycoprotein [34]. Data from these *in vivo/vitro* functional studies gave some support to the hypothesis that the 5/5 homozygous genotype might confer more resistance to HIV-1 infection. However, these associations were far from conclusive. More functional *in vivo* studies are needed.

Although meta-analysis is a powerful statistical method, inherent limitations of this study should be addressed. First, we only included the studies written in English and Chinese, and the related reports in other languages were not included, which might bias our conclusion in this study. Second, publication bias could not be excluded though the test showed negative results. The studies reporting significant associations between certain genotypes and reduced susceptibility to HIV infection would be more readily published while the studies with nonsignificant associations would be more difficult to publish. Third, most of the studies were conducted using Asian population groups. In the stratified analysis by ethnicity, there was only one study performed with Africans and two studies with European Americans, each of which had a sample size too small to achieve enough statistical power to obtain significant observations. In fact, many association studies showed different results for different populations. Thus, further studies are warranted in other ethnic populations to evaluate the possible ethnic differences of the VNTR polymorphism and HIV-1 susceptibility. Fourth, gene-gene and gene-environment interactions may influence host susceptibility to HIV-1 infection. In fact, many genes have been proven to influence HIV-1 infection risk, but we did not have enough data to eliminate these interfering factors. Fifth, as most studies did not mentioned about potential population stratification of patients and controls samples, we cannot rule out a role of population structure in the observed association. Finally, further stratified analyses of patients and HESN individuals by infection exposure routes (sexual contact, intravenous drug use, etc.) could not be performed because the data detailing the infection route for the HIV-1 patients were lacking. Because DC-SIGNR is expressed at relatively lower levels in tissues *in vivo* [35], the association between the VNTR polymorphism and HIV-1 infection risk by different infection routes might be different.

Humans show remarkable variation in vulnerability to infection by HIV-1 and especially in the clinical outcomes after infection. Understanding why some people establish and maintain effective control of HIV-1 and others do not is a priority in the effort to develop new treatments for HIV/AIDS. To our knowledge, this is the first meta-analysis to assess the relationship between the DC-SIGNR VNTR polymorphism and HIV-1 infection risk. Our results showed that the 5/5 homozygous genotype was associated with resistance to HIV-1 in HIV-1 exposed seronegative subjects (HESN), especially in the Asian population. Future studies in different ethnic populations and with clear infection routes should be performed to evaluate these associations.

## Supporting Information

Table S1 **The breakdown of HESN controls in the included studies of this meta-analysis according the routine of exposure.**
(DOC)

Table S2 **Meta-analysis of association between the DC-SIGNR VNTR polymorphism and HIV-1 infection.**
(DOC)
