# Supplementary material for: The VNTR Polymorphism of the DC-SIGNR Gene and Susceptibility to HIV-1 Infection: A Meta-Analysis
Source: PLoS One. 2012 Sep 5;7(9):e42972. doi: 10.1371/journal.pone.0042972 (PMC3434151; doi:10.1371/journal.pone.0042972)
Supplement: Table S1 — The breakdown of HESN controls in the included studies of this meta-analysis according the routine of exposure. (DOC) [file pone.0042972.s001.doc]

| Studies | Total Number of HESN controls | Commercial Sex Workers | Discordant Couples | Intravenous Drug Users | Mother-to-Child Transmission | Others^a^ |
| --- | --- | --- | --- | --- | --- | --- |
| Liu H | 217 | 94 |  |  |  | 123 |
| Rathore A | 47 | 47 |  |  |  |  |
| Chaudhary O | 150 |  |  |  |  | 150 |
| Wichukchinda N | 102 |  | 102 |  |  |  |
| Wang H | 468 | 203 | 26 | 239 |  |  |
| Wang XH | 52 | **+** | **+** |  |  |  |
| Boily-Larouche G | 99 |  |  |  | 99 |  |

Table S1 The breakdown of HESN controls in the included studies of this meta-analysis according the routine of exposure.

Note:

a: The others referred to the source of the HESN controls was the mixture of the commercial sex workers, discordant couples, intravenous drug users and mother-to-child transmission. However, the exact number in each group was not given in the original paper.

+ : HESN controls in the Wang XH’s study were recruited from the commercial sex workers and discordant couples, but the exact number of each groups were not given in the original paper.
