# Supplementary material for: The VNTR Polymorphism of the DC-SIGNR Gene and Susceptibility to HIV-1 Infection: A Meta-Analysis
Source: PLoS One. 2012 Sep 5;7(9):e42972. doi: 10.1371/journal.pone.0042972 (PMC3434151; doi:10.1371/journal.pone.0042972)
Supplement: Table S2 — Meta-analysis of association between the DC-SIGNR VNTR polymorphism and HIV-1 infection. (DOC) [file pone.0042972.s002.doc]

Table S2 Meta-analysis of association between the DC-SIGNR VNTR polymorphism and HIV-1 infection.

| **Table S2-A** Summary of primary analysis of allelic association and HIV-1 infection. | | | |  |
| --- | --- | --- | --- | --- |
| **Study Group** | **7 allele vs. other alleles** | **5 allele vs. other alleles** | **6 allele vs. other alleles** | **9 allele vs. other alleles** |
|  | OR (95% CI) *P*^a^ | OR (95% CI) *P* | OR (95% CI) *P* | OR (95% CI) *P* |
| **Total** |  |  |  |  |
| HC^b^ as control | 0.93(0.85,1.02) 0.27 | 1.03(0.93,1.15 ) 0.22 | 0.66(0.37,1.19) <0.00001 | 1.02(0.87,1.19) 0.73 |
| HESN^c^ as control | 1.16(0.94,1.43) 0.008 | **0.84(0.73,0.96) 0.18** | 0.79(0.26,2.41) <0.00001 | 0.91(0.75,1.10) 0.11 |
| **Subgroup by Ethnicity** |  |  |  |  |
| **Asian** |  |  |  |  |
| HC as control | 0.98(0.86,1.12) 0.19 | 0.93(0.79.1.10) 0.39 | 0.84(0.61,1.14) 0.96 | 0.66(0.30,1.41) <0.00001 |
| HESN as control | 1.11(0.84,1.47) 0.01 | **0.84(0.71,1.00) 0.20** | 1.04(0.76,1.42) 0.11 | 0.84(0.21,3.35) <0.00001 |
| **European** |  |  |  |  |
| HC as control | 0.89(0.78,1.01) 0.67 | 1.12(0.97,1.29) 0.19 | 1.09(0.91,1.30) 0.32 | 0.63(0.29,1.38) 0.17 |
| **Subgroup by Sample Size** |  |  |  |  |
| **>200 subjects** |  |  |  |  |
| HC as control | 0.91(0.83,1.00) 0.43 | 1.05(0.94,1.17 ) 0.22 | 1.03(0.88,1.20) 0.66 | 0.65(0.33,1.28) <0.00001 |
| HESN as control | 1.14(0.87,1.49) 0.002 | **0.82(0.68,0.98) 0.21** | 1.05(0.74,1.47) 0.19 | 0.64(0.18,2.28) <0.00001 |
| **<200 subjects** |  |  |  |  |
| HC as control | 1.33(0.87,2.04) ── | 0.77(0.46,1.29) ── | 0.80(0.34,1.85) ── | 0.70(0.33,1.49) ── |
| HESN as control | 1.25(0.90,1.73) 0.82 | 1.83(0.15,22.07) 0.08 | 0.65(0.44,0.97) 0.65 | 2.32(0.64,8.32) ── |

|  |  |  |  |  |  |  |
| --- | --- | --- | --- | --- | --- | --- |
|  |  |  |  |  |  |  |
|  |  |  |  |  |  |  |
|  |  |  |  |  |  |  |
|  |  |  |  |  |  |  |
|  |  |  |  |  |  |  |

| **Study Group** | **Homozygous vs. heterozygous** | **5/5 vs. other genotypes** | **7/7 vs. other genotypes** | **5/7 vs. other genotypes** | **6/7 vs. other genotypes** | **7/9 vs. other genotypes** |
| --- | --- | --- | --- | --- | --- | --- |
|  | OR (95% CI) *P*^a^ | OR (95% CI) *P* | OR (95% CI) *P* | OR (95% CI) *P* | OR (95% CI) *P* | OR (95% CI) *P* |
| **Total** |  |  |  |  |  |  |
| HC^b^ as control | 0.94 (0.83,1.07) 0.14 | 0.90(0.71,1.14) 0.23 | 0.95(0.83,1.08) 0.16 | 0.99(0.85,1.16) 0.52 | 0.86(0.68,1.07) 0.80 | 0.87(0.58,1.31) 0.02 |
| HESN^c^ as control | 1.04 (0.75,1.45) 0.002 | **0.68(0.50,0.93) 0.21** | 1.25(0.88,1.76) 0.001 | 0.87(0.63,1.21) 0.03 | 0.90(0.70,1.17) 0.57 | 1.26(0.96,1.66) 0.72 |
| **Subgroup by Ethnicity** |  |  |  |  |  |  |
| **Asian** |  |  |  |  |  |  |
| HC as control | 0.94(0.72,1.24) 0.06 | 0.71(0.48,1.05) 0.44 | 1.00(0.78,1.29) 0.08 | 0.96(0.77,1.20) 0.41 | 0.76(0.51,1.13) 0.75 | 1.08(0.84,1.39) 0.16 |
| HESN as control | 0.98(0.66,1.44) 0.01 | **0.58(0.39,0.85) 0.34** | 1.12(0.76,1.64) 0.02 | 0.91(0.60,1.36) 0.03 | 0.81(0.54,1.22) 0.34 | 1.23(0.93,1.63) 0.57 |
| **European** |  |  |  |  |  |  |
| HC as control | 0.97(0.81,1.17 )0.84 | 1.05(0.77,1.43) 0.11 | 0.92(0.76,1.12) 0.44 | 1.06(0.87,1.30) 0.51 | 0.96(0.75,1.22) 0.46 | 0.74(0.19,2.81) 0.09 |
| **Subgroup by Sample Size** |  |  |  |  |  |  |
| **>200 subjects** |  |  |  |  |  |  |
| HC as control | 0.92(0.81,1.08) 0.17 | 0.91(0.71,1.16) 0.16 | 0.93(0.81,1.06) 0.20 | 1.00(0.85,1.18) 0.40 | 0.86(0.68,1.08) 0.68 | 0.89(0.57,1.40) 0.01 |
| HESN as control | 1.03(0.68,1.56) 0.0004 | **0.69(0.50,0.94) 0.13** | 1.24(0.79,1.94) 0.0003 | 0.87(0.59,1.27) 0.01 | 0.94(0.69,1.27) 0.47 | 1.24(0.94,1.64) 0.62 |
| **<200 subjects** |  |  |  |  |  |  |
| HC as control | 1.42(0.80,2.53) ── | 0.72(0.21,2.48) ── | 1.42(0.80,2.53) ── | 0.90(0.46,1.78) ── | 0.87(0.27,2.81) ── | 0.66(0.24,1.86) ── |
| HESN as control | 1.07(0.69,1.67) 0.37 | 0.63(0.16,2.43) ── | 1.30(0.84,2.01) 0.92 | 0.82(0.39,1.74) 0.38 | 0.80(0.47,1.36) 0.36 | 1.86(0.37,9.30) ── |

**Table S2-B** Summary of other post-hoc analysis with the genetic models based on different genotypes.

| Note: a: *P* value of Q-test for heterogeneity test | |
| --- | --- |
| b: healthy normal controls |  |
| c: HIV-1 exposed but seronegative controls | |
| The bold values mean that their association is significant. | |
